# Evaluating Large Language Models to Support Dementia Caregivers: Identifying Opportunities for Improvement

**DOI:** 10.1093/geroni/igaf122.4109

**Published:** 2025-12-31

**Authors:** Kyeung Mi Oh, Sungsoo Hong, Ziwei Zhu, Huayu Zhou, Jung Ah Lee

**Affiliations:** George Mason University, Fairfax, Virginia, United States; George Mason University, Fairfax, Virginia, United States; George Mason University, George Mason University, Virginia, United States; George Mason University, George Mason University, Virginia, United States; University of California, Irvine, University of California, Irvine, California, United States

## Abstract

Awareness and access to the dementia caregiving resources is crucial for informal caregivers of people with early-stage dementia. Large language models (LLMs) offer easy access to caregiving information, but the risks, challenges, and ways to improve LLM-generated responses remain understudied. This mixed methods study evaluated LLMs, including the baseline ChatGPT-4o model and an enhanced version refined through prompt engineering grounded in health science and gerontology literature, to support informal dementia caregivers. This study aimed to assess key factors influencing preferred responses from LLMs and to identify related risks and challenges, thereby informing opportunities for improvement. Surveys and interviews with 12 stakeholders, including 10 healthcare professionals and 2 caregivers, were conducted to assess model responses to questions commonly asked by caregivers. The responses were assessed using validated multidimensional measures based on a human evaluation framework for LLMs in healthcare. Survey results showed the enhanced ChatGPT-4o model scored significantly higher in actionability, relevance, and satisfaction than the baseline ChatGPT-4o version. However, no significant differences were observed between the models in accuracy, understanding, intelligibility, trust, safety, or potential harm. Key themes from interview data influencing preferred responses included wordiness, in-depth content, empathy, actionability, accuracy, relevance, and bias. Overall, while both models’ responses were perceived as overly verbose, the enhanced model provided more comprehensive and caregiver-centered information than the baseline ChatGPT-4o. These findings suggest that LLMs can deliver more practical and satisfying guidance for dementia caregiving. Incorporating domain-specific frameworks into model design may enable scalable, evidence-based support for caregivers in real-world settings.

